# *HLA-H*: Transcriptional Activity and HLA-E Mobilization

**DOI:** 10.3389/fimmu.2019.02986

**Published:** 2020-01-17

**Authors:** François Jordier, Delphine Gras, Maria De Grandis, Xavier-Benoît D'Journo, Pascal-Alexandre Thomas, Pascal Chanez, Christophe Picard, Jacques Chiaroni, Julien Paganini, Julie Di Cristofaro

**Affiliations:** ^1^Aix-Marseille University, CNRS, EFS, ADES, “Biologie des Groupes Sanguins”, Marseille, France; ^2^Etablissement Français du Sang PACA Corse, Marseille, France; ^3^Aix-Marseille University, INSERM, INRA, C2VN, Marseille, France; ^4^Department of Thoracic Surgery, North Hospital, Aix-Marseille University & Assistance Publique-Hôpitaux de Marseille, Marseille, France; ^5^Clinique des Bronches, Allergie et Sommeil, North Hospital, Assistance Publique-Hôpitaux de Marseille, Marseille, France; ^6^Xegen, Gémenos, France

**Keywords:** HLA-H, HLA-E, expression, signal peptide, pseudogene

## Abstract

Little attention is paid to pseudogenes from the highly polymorphic HLA genetic region. The pseudogene *HLA-H* is defined as a non-functional gene because it is deleted at different frequencies in humans and because it encodes a potentially non-functional truncated protein. However, different studies have shown HLA-H transcriptional activity. We formerly identified 13 novel *HLA-H* alleles, including the H*02:07 allele, which reaches 19.6% in East Asian populations and encodes a full-length HLA protein. The aims of this study were to explore the expression and possible function of the HLA-H molecule. HLA-H may act as a transmembrane molecule and/or indirectly via its signal peptide by mobilizing HLA-E to the cell surface. We analyzed *HLA-H* RNA expression in Peripheral Blood Mononuclear Cells (PBMC), Human Bronchial Epithelial Cells (HBEC), and available RNA sequencing data from lymphoblastoid cell lines, and we looked to see whether HLA-E was mobilized at the cell surface by the HLA-H signal peptide. Our data confirmed that *HLA-H* is transcribed at similar levels to *HLA-G*. We characterized a hemizygous effect in *HLA-H* expression, and expression differed according to *HLA-H* alleles; most interestingly, the HLA-H*02:07 allele had the highest level of mRNA expression. We showed that HLA-H signal peptide incubation mobilized HLA-E molecules at the cell surface of T-Lymphocytes, monocytes, B-Lymphocytes, and primary epithelial cells. Our results suggest that HLA-H may be functional but raises many biological issues that need to be addressed.

## Introduction

The highly polymorphic Human Leukocyte Antigen (HLA) genetic region encompasses many pseudogenes; little attention is paid to these compared to class I and II genes involved in clinical fields, such as immune diseases, anthropology matters, and the early migration of *Homo sapiens* (1, 2). The pseudogene *HLA-H* is located at 55 Kbp from the telomeric side of *HLA-A* and, due to their high similarity, these genes were described as sharing a recent ancestor (3–5).

*HLA-H* was defined as a non-functional gene because of its deletion from chromosomes carrying HLA-A*23/^*^24 alleles (6–11), but also because its amino acid sequence predicted a potentially non-functional truncated protein.

In a previous study, we identified 13 novel *HLA-H* alleles, which showed unexpected genetic diversity, with a total of 25 second-field alleles (10). Among these, H*02:07 and H*02:14 potentially encode complete transmembrane HLA proteins; while the H*02:14 allelic frequency was very low, H*02:07 displayed global worldwide frequencies of 8.6% that reached 19.6% in East Asian populations (10).

Functional implications of a putative HLA-H protein, however, remain difficult to explore as, to date, there is no validated HLA-H antibody. Nevertheless, the transcriptional activity of this gene has been assessed in different studies (12–14).

Like the non-classical class I molecules, HLA-G, -E, and -F, which display immune response activation and inhibition (15–21) HLA-H may be tolerogenic and participate in immune homeostasis. In cases where the immune system is challenged, the absence of HLA-H might lessen tolerogenicity; in Lung Transplant patients (LTx), the HLA-G*01:04 allele, in Linkage Disequilibrium (LD) with HLA-H*deletion, was associated with impaired long-term survival, increased Chronic Lung Allograft Dysfunction (CLAD) occurrence, and the production of *de novo* Donor Specific Antigen (DSA) (22). The impaired outcome associated with HLA-G*01:04 remains unclear as the HLA-G^*^01:04 protein, which differs from G^*^01:01 in its peptide anchor profile, increased protection from Natural Killer cells (NK) lysis compared to other alleles (in cytotoxicity assays with K562 cells) (23). Several causes may be considered, however, such as antigenicity elicited by HLA-G^*^01:04 or reduced HLA-G expression in HLA-G*01:04 carriers; however, it could also be due to the absence of *HLA-H* with the HLA-G*01:04 haplotype.

HLA-H may act like HLA-G, -E, and -F, both directly as a transmembrane molecule and/or indirectly via its signal peptide by mobilizing HLA-E to the cell surface. HLA-E, which regulates NK and cytotoxic T-lymphocyte cells via the inhibitory receptor CD94/NKG2 (16, 18, 19), is transcribed in most tissues (24) but is mobilized to the cell surface by signal peptides from HLA Ia, HLA-G, and peptide ligands from stress proteins and viruses (25, 26).

The aims of the present study were to explore the expression and possible function of the HLA-H molecule. Lacking a validated tool to analyze the putative HLA-H transmembrane protein, we analyzed *HLA-H* RNA expression in Peripheral Blood Mononuclear Cells (PBMC), Human Bronchial Epithelial Cells (HBEC), and available RNA sequencing data from lymphoblastoid cell lines, and we looked to see whether HLA-E molecules were mobilized at the cell surface by the HLA-H signal sequence.

## Materials and Methods

### Primary Cells

Peripheral Blood Mononuclear Cells (PBMC) were obtained from Ethylene Diamine Tetra Acetate-anticoagulated (EDTA) peripheral blood samples from healthy donors. The donations were collected in accordance with the French blood donation regulations and ethics and with the French Public Health Code (article L.1221-1).

Human Bronchial Epithelial Cells (HBEC) were obtained from human transplant donor lungs deemed unsuitable for transplantation and donated to medical research. The ethics committees of the institutions involved approved this study (CERC-SFCTCV-2018-5-6-9-8-32-DjXa). Primary human bronchial epithelial cells were isolated by protease digestion of human airways, and cells were cultivated under Air-Liquid Interface (ALI) conditions, as previously described (27). HBEC were maintained in culture for 21 days to obtain a differentiated cell population with a mucociliary phenotype.

K562 (ACC86) cell lines, used as the negative control for HLA expression, were obtained from the German Collection of Microorganisms and Cell Cultures (Leibniz Institute DSMZ, Germany).

### HLA-H Transcriptional Expression

#### HLA-H Transcriptional Expression in PBMC and Bronchial Epithelial Cells

*HLA-H* transcriptional expression was investigated in PBMC and HBEC from healthy donors (*N* = 5 and *N* = 8, respectively). K562 cells (ACC86) were used as the negative control. Total RNA was isolated using the RNeasy kit (Qiagen, France). cDNA was reverse transcribed using Superscript III Reverse Transcriptase (Invitrogen), and Real-time PCR analyses were performed using TaqMan technology (Life Technologies) as previously described (28). The primers/probes were designed using the Primer 3 v.0.4.0 program (http://bioinfo.ut.ee/primer3-0.4.0/primer3/) to specifically target *HLA-H* as checked with the Human Genome Browser (http://genome.ucsc.edu/) (29) and IPD-IMGT/HLA database 3.37.0 (30) (Supplementary Table 1). HLA-G and HLA-E were investigated as previously described (28), and *ACTB* (actinβ) was used as an endogenous control (ACTB Hs99999903_m1, Invitrogen).

Each experiment was carried out in duplicate, and average Ct was calculated with StepOne 2.1 software (Invitrogen), but Ct duplicates with a standard deviation above 0.5 were excluded.

#### HLA-H Transcriptional Expression in Cell Line From the 1000 Genomes Project

*HLA-H* expression was also investigated in RNA-sequencing data from 464 lymphoblastoid cell lines from the 1000 Genomes Project (31).

RNA sequencing data were analyzed using PolyPheMe software specially designed for HLA NGS data analysis (Xegen, France) for which accuracy was assessed at 99.3% (10, 32, 33). This software is based on a specific strategy to avoid bias raised by the use of a unique genome as a reference for HLA NGS data mapping (34–36). First, RNAseq reads were selected for their specificity for *HLA* class I alleles. Then, all reads assigned to *HLA* class I were filtered out for their specificity for *HLA Ia* (*A, B, C*), *Ib* (*E, F, G*), and pseudogenes that showed the highest homology with *HLA-H*. Lastly, remaining reads were selected for their specificity for *HLA-H* using all exon sequences. The analysis was based on the 25 *HLA-H* alleles described in the IPD-IMGT/HLA database 3.37.0 (30), including the new alleles described in (10).

### HLA-E Mobilization by HLA-H Signal Peptide

HLA-E mobilization by a HLA-H signal peptide was investigated in PBMC and HBEC using flow cytometry analysis.

Lyophilized signal peptides from HLA-H and from positive controls [HLA-G, HLA-B15, and cytomegalovirus (CMV) (37)] were purchased from Invitrogen with 95% purity. Dimethylsulfoxyde (DMSO) and Neuromedin peptide (Sigma Aldrich) were used as reference and negative controls, respectively. All peptides were solubilized in DMSO at 50 mM. Peptide sequences are presented in Supplementary Table 2.

Cells were incubated as described in (37) with each peptide at a final concentration of 500 μM. Cells were incubated with the equivalent volume of peptide solvent (DMSO) as reference. PBMC with known *HLA-E* genotype were incubated at 37°C for 1, 2, 4, and 6 h. For HBEC, incubation at both interfaces (air and liquid) was performed at 37°C for 16 h. Each experiment was carried out in duplicate.

HLA-E expression at the cell surface was assessed by flow cytometry using a Mouse IgG1 antibody clone 3D12-PE (Invitrogen). The isotype control was a Mouse IgG1 antibody clone 679.1Mc7-PE (Beckman Coulter). Data were acquired on a FACSCalibur machine (BD Biosciences) and analyzed with BD FACSDiva software 6.1.2.

PBMC subtypes for HLA-E expression and overexpression after incubation with HLA-H were characterized with an antibody panel that included CD3-vioBlue (Miltenyi), CD19-AF700 (Biolegend), CD14-FITC (Biolegend), and viability Dye (eFluor506) (eBioscience) with or without a 3D12 antibody. Data were acquired on a Cytoflex machine (Beckman Coulter).

### Statistical Analyses

All association and correlation tests were performed with GRAPH PAD Prism 5 software (California USA). Differences between two modalities were tested using a Mann–Whitney *U* test. A Kruskal–Wallis one-way ANOVA followed by a Dunn *post-hoc* test was used to test more than two modalities. Q-PCR results were expressed as dCt (delta of cycle threshold, expression normalized by *ACTB* endogenous gene) with median and range [min, max]. RNA seq data from 464 lymphoblastoid cell lines from the 1000 Genomes Project were presented as the number of reads. HLA-E protein expression without peptide incubation was estimated as a percentage of expressing cells (cells gated in R2 and defined according to isotype control staining). The HLA-E mobilization at the cell surface upon peptide incubation was estimated and compared to incubation with a solvent (DMSO). Mobilization was assessed by the number of cells expressing HLA-E compared with DMSO (% gated in M2: cells with higher fluorescence than those incubated with DMSO), and by the ratio M2 Mean Fluorescence Intensity (MFI) median (cells incubated with peptide with gated cells in M2 >1%)/M1 MFI median (cells incubated with DMSO).

## Results

### *HLA-H* mRNA Is Expressed in PBMC and HBEC

**Figure 1:**
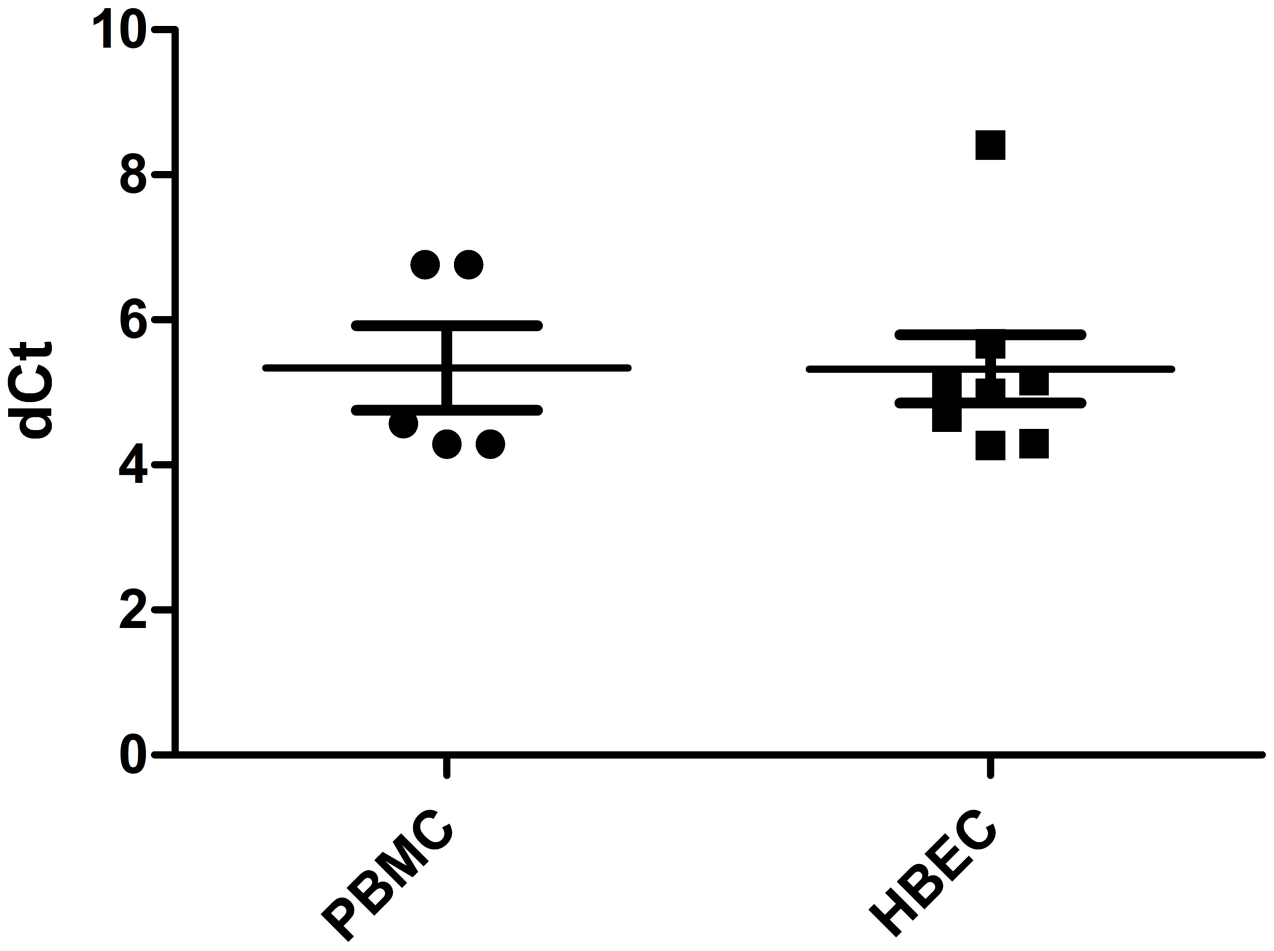
HLA-H expression in PBMC and HBEC (dCt: delta of cycle threshold, expression quantified by Q-PCR normalized by ACTB endogenous gene).

*HLA-H* transcriptional expression was measured using Real Time PCR. *HLA-H* primer/probe efficiency, estimated by 10-fold dilution of HBEC mRNA assays, was 2.05; no signal was observed with the gDNA assay or in K562 cells. All PBMC and HBEC samples expressed *HLA-H* [5.334 (4.291–6.758) and 5.322 (4.268–8.410), respectively] (Figure 1).

In HBEC, *HLA-H* transcriptional expression was similar to that of *HLA-G*, both were lower than that of *HLA-E* (*p* < 0.0001) (Supplementary Figure 1).

### *HLA-H* mRNA Expression Is Lower in Hemizygous *HLA-H* Samples

*HLA-H* expression investigated in RNA-sequencing data from the 1000 Genomes Project (31) showed *HLA-H*-specific reads for 464 samples. As *HLA-H* is deleted from chromosomes bearing HLA-A*23/*24 alleles, two samples bearing HLA-A*24:02/*24:02* and HLA-A*23:01/*24:02* showed 22 and 203 *HLA-H* reads, respectively, representing background noise. No attempt was made to correct this mismapping bias that is inherent to HLA NGS data mapping (34–36).

*HLA-H* expression in samples displaying one HLA-A*23 or ^*^24 allele had statistically significant lower *HLA-H* reads [*N* = 96, 192.6 (22–1,049)] than samples with no HLA-A*23 or ^*^24 allele [*N* = 365, 334.8 (39–2,135) the outer value at 7,924 was excluded from analysis; *p* < 0.0001] (Supplementary Figure 2). This hemizygous effect was confirmed independently from allelic effect in genetically homogeneous samples with sufficient overall size: H*01:01/HLA-A*23 or ^*^24 samples that displayed statistically significant lower *HLA-H* reads than H*01:01/01:01 samples [*N* = 29, 126.2 (46–227) vs. *N* = 37, 222.5 (78–623); *p* = 0.0002] (Supplementary Figure 3).

### *HLA-H* mRNA Expression Varies According to *HLA-H* Alleles

**Figure 2:**
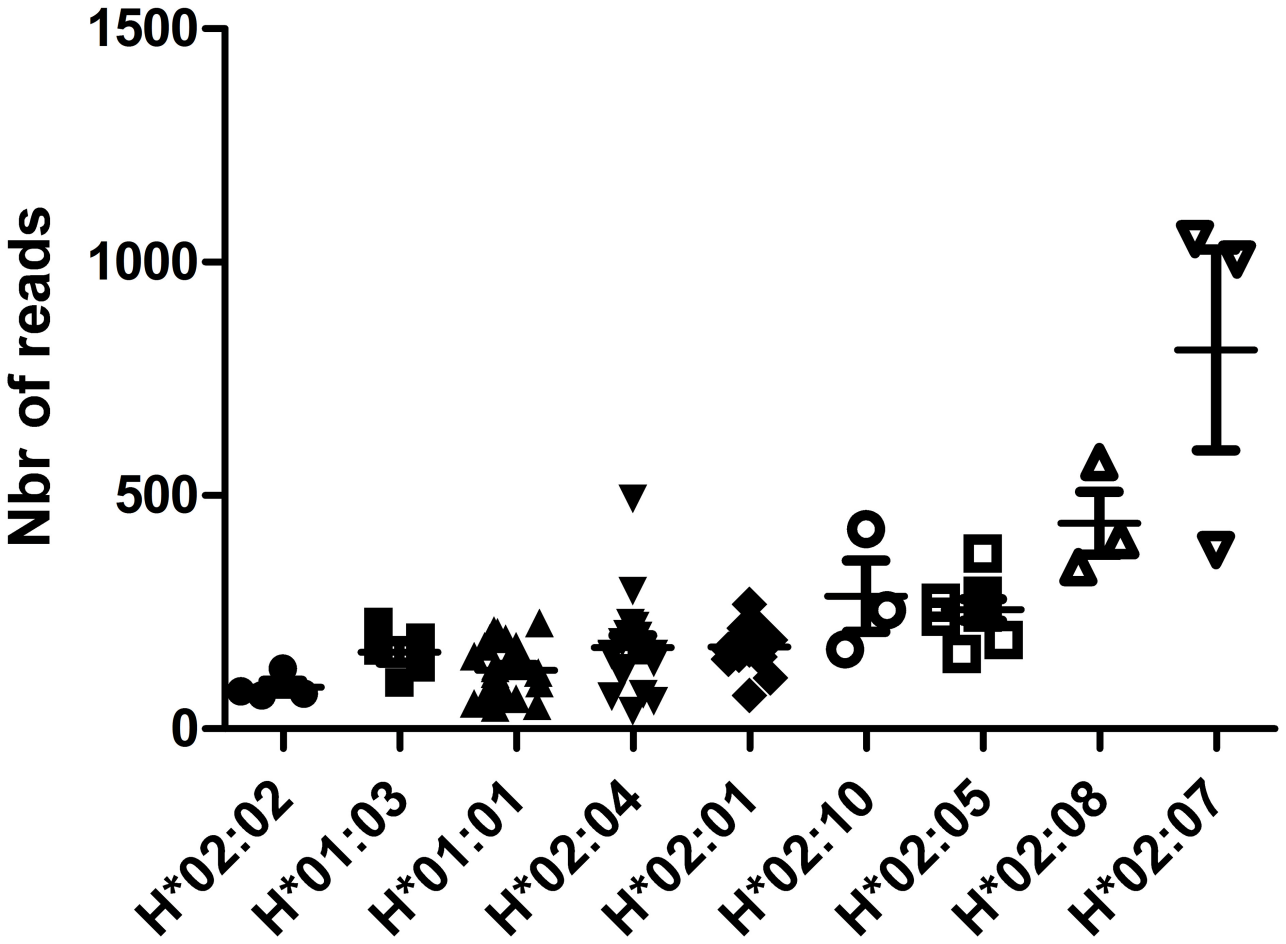
HLA-H reads according to HLA-H alleles in hemizygous samples for HLA-H in RNA-sequencing data from the 1000 Genomes Project.

Expression of *HLA-H* mRNA differed according to *HLA-H* alleles in RNA-sequencing data from the 1000 Genomes Project; hemizygous samples (bearing HLA-A*23 or ^*^24 alleles) showed low-expression alleles {HLA-H*02:02 [*N* = 4, 89.50 (72–129)], H*01:03 [*N* = 5, 164.8 (99–229)], H*01:01 [*N* = 29, 126.2 (46–227)], H*02:04 [*N* = 17, 174.6 (38–493)], H*02:01 [*N* = 13, 175.2 (72–267)], medium-expression alleles {H*02:10 [*N* = 3, 284.3 (171–428)], H*02:05 [*N* = 8, 255.1 (161–376)], H*02:08 [*N* = 3, 441 (347–572)]}, and a high-expression allele {H^*^02:07 [*N* = 3, 812.3 (383–1049)]}; *p* < 0.0001 (Figure 2). This difference was confirmed in homozygous samples of sufficient overall size: low-expression alleles {H*01:01/*01:01 [*N* = 37, 222.5 (78–623)], H*02:04/*02:04 [*N* = 18, 211.3 (39–537)], H*02:01/*02:01 [*N* = 6, 181.8 (53–283)]} and a medium-expression allele {H*02:05/*02:05 [*N* = 6, 487.5 (314–718)]}; *p* = 0.0051 (Supplementary Figure 4). H*02:070 of a higher expression was confirmed in samples bearing H*01:01 and H*01:01/*02:07 [*N* = 20, 886.8 (353–1,455)] vs. H*01:01/*01:01 [*N* = 37, 222.5 (78–623)]; *p* < 0.0001 (Supplementary Figure 5).

### HLA-E Molecule Is Mobilized at PBMC and HBEC Surface by HLA-H Signal Peptide

The expression of the HLA-E protein at the cell surface was studied using flow cytometry. All PBMC and HBEC expressed HLA-E without peptide incubation [3D12 (anti-HLA-E) staining compared to isotype control]. In PBMC, HLA-E*01:03 homozygous individuals expressed more HLA-E than heterozygous individuals and E*01:01 homozygous individuals [74.88 (59.39–85.68); 59.79 (51.34–65.30); 39.55 (35.76–44.90), respectively, *p* = 0.0015] (Supplementary Figure 6).

**Figure 3:**
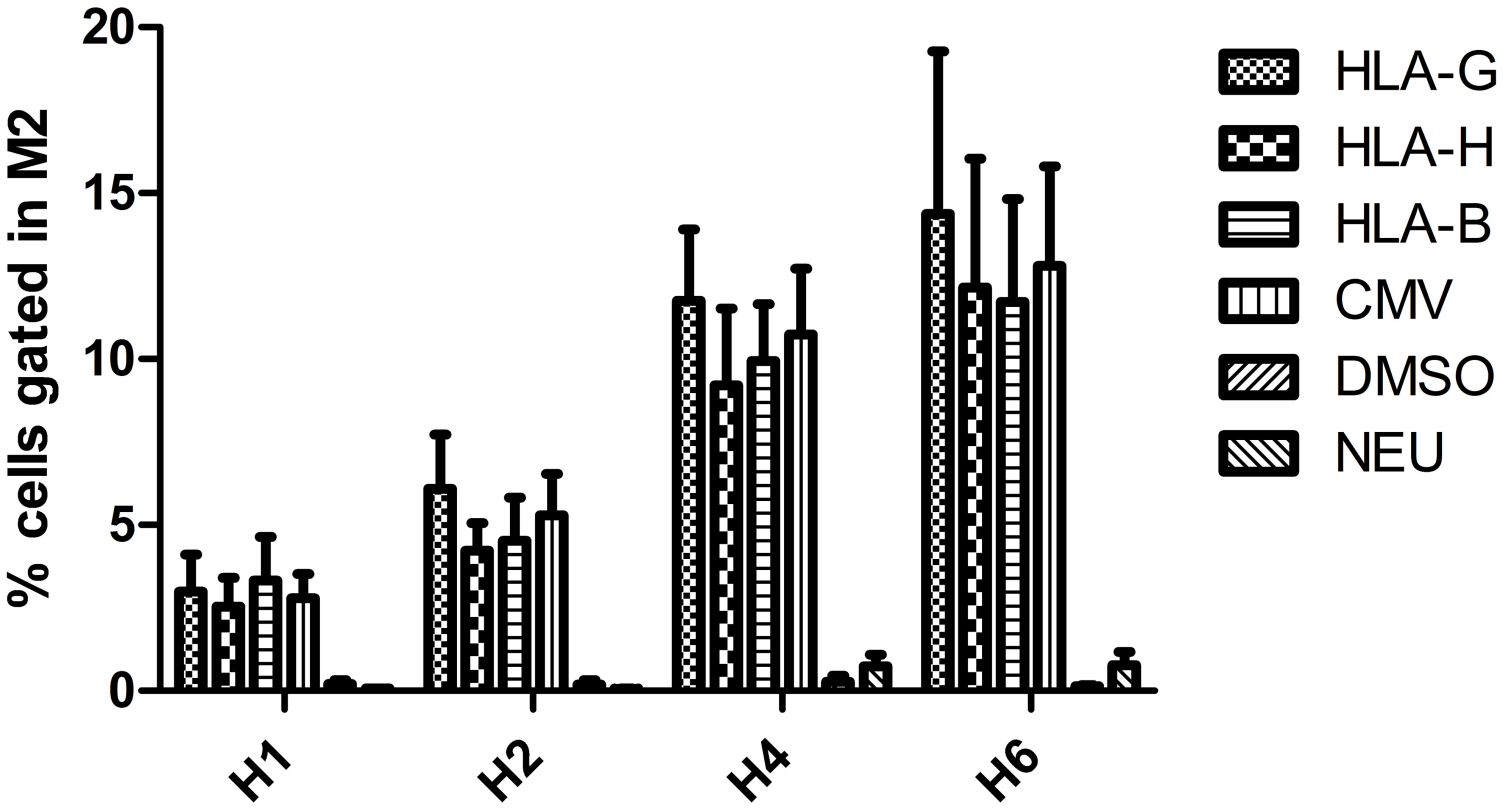
Percentage of PBMC overexpressing HLA-E compared with DMSO (defining M2) after 1, 2, 4, and 6 h of peptide incubation.

Incubation with CMV, HLA-B15, HLA-G, and HLA-H peptides significantly increased the number of cells expressing HLA-E after 4 and 6 h of incubation (*p* < 0.01), whereas incubation with the negative control Neuromedin showed no effect (Figure 3). In Neuromedin or DMSO assays, M2-gated cells never reached 1%.

**Figure 4:**
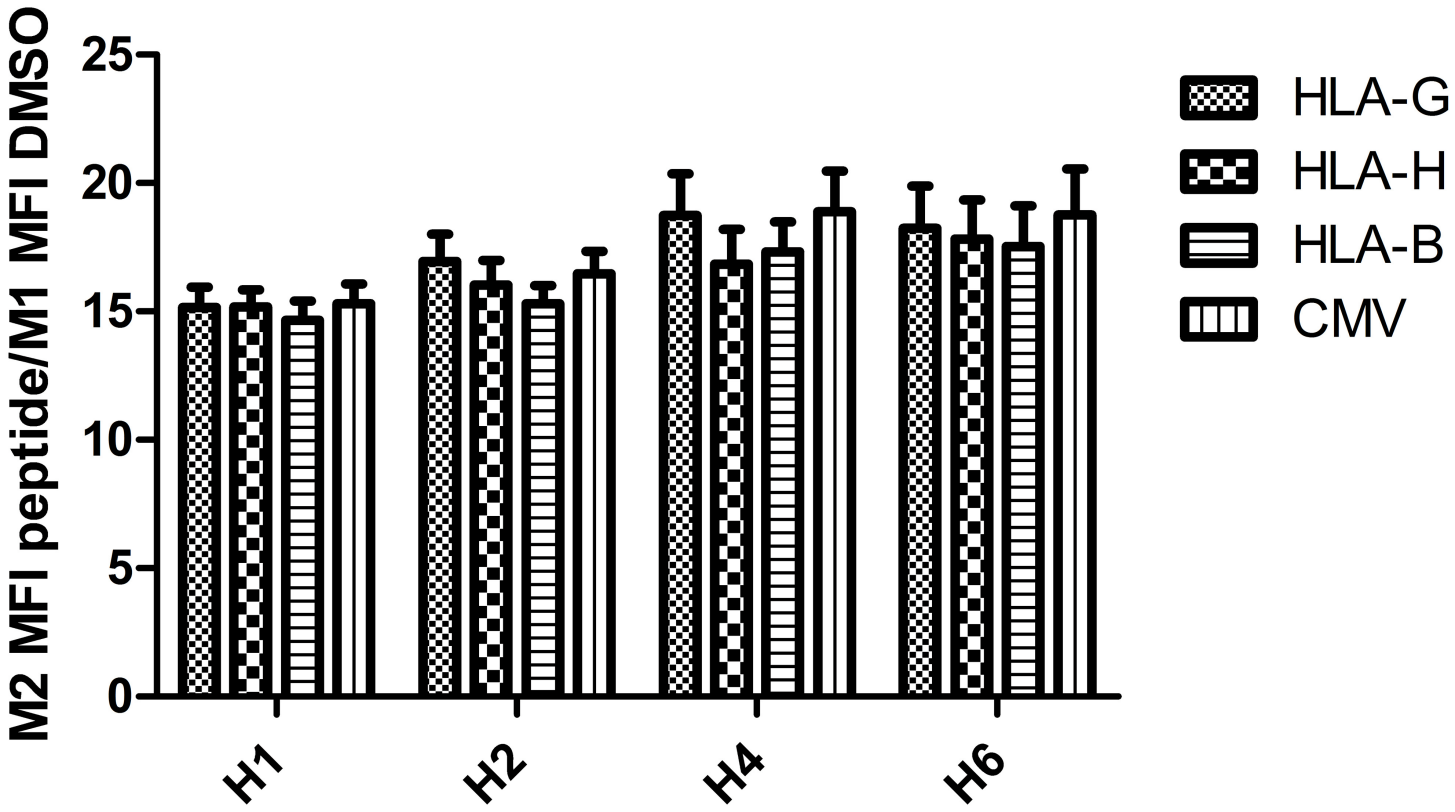
PBMC HLA-E overexpression after 1, 2, 4, and 6 h of peptide incubation (gated cells in M2 >1%) compared with DMSO.

Intensity of HLA-E mobilization at the cell surface was equivalent after incubation with an HLA-H peptide and with positive control peptides: no difference was observed between CMV, HLA-B15, HLA-G, and HLA-H (*p* = 0.469). Incubation duration significantly increased expression (*p* = 0.0014); the highest expression was reached after 4 h of incubation (Figure 4).

PBMC cell subtype analysis showed that LT, monocytes, and LB overexpressed HLA-E after 4 h of HLA-H peptide incubation (Supplementary Figure 7).

In HBEC that were tested with one peptide control because of HBEC availability, HLA-E mobilization with HLA-H, and CMV peptides showed no statistical difference and were effective after 16 h of peptide incubation (*p* = 0.0273) compared to DMSO (Supplementary Figure 8).

## Discussion

HLA pseudogenes have scarcely been studied and are poorly investigated, but some of them may still be functional. *HLA-H* was shown to be transcribed (12–14), and we formerly described unexpected worldwide genetic diversity (10). In different clinical studies, the HLA-G*01:04 haplotype, in which *HLA-H* was deleted, was associated with an impaired outcome (22, 38, 39). The full-length HLA-H protein showed similar domains to non-classical class I molecules (10), HLA-G, -E, and -F, molecules, which displayed immune response activation and inhibition (15–21). Furthermore, the HLA-H signal peptide (MVLMAPRTLLLLLSGALALTQTWA) was almost identical to that of HLA-A (MAVMAPRTLLLLLSGALALTQTWA), except that there was a Valine in the second position as in HLA-G (MVVMAPRTLFLLLSGALTLTETWA), and there was a specific amino acid (Val>Leu) in the third position, compared to other HLA class I proteins (40).

We thus hypothesized that HLA-H may have tolerogenic activity either as a transmembrane molecule and/or via its signal peptide by mobilizing HLA-E to the cell surface.

In this study, as there is still no validated anti-HLA-H antibody, we aimed to confirm *HLA-H* RNA expression in different primary cells and also to see whether the HLA-H signal peptide could mobilize HLA-E to the cell surface in a similar way to other HLA peptides.

We analyzed *HLA-H* RNA expression in Peripheral Blood Mononuclear Cells (PBMC), Human Bronchial Epithelial Cells (HBEC), and in RNA-sequencing data from 464 lymphoblastoid cell lines from the 1000 Genomes Project (31).

Our data support that *HLA-H* is transcribed in blood mononuclear cells and in primary epithelial cells (12–14). In HBEC, its expression is similar to that of *HLA-G*. RNA-sequencing data from lymphoblastoid cell lines from the 1000 Genomes Project allowed us to show a hemizygous effect in the expression of *HLA-H* and to characterize different levels of expression according to *HLA-H* alleles; most interestingly, the HLA-H*02:07 allele, which potentially encodes a full-length protein, presents the highest level of mRNA expression. *HLA-H* expression results from RNA-sequencing data, but this should, however, be taken with precaution and needs further confirmation; a part of the reported expression level, notably in the low-expression alleles, may be due to mismapped reads, as observed in the HLA-A*23 and/or ^*^24 samples and as reported in different studies dealing with HLA NGS mapping (34–36).

We then analyzed HLA-E mobilization at the cell surface by peptides from the HLA-H signal sequence. Physiologically, HLA-E is expressed at the surface of endothelial cells, T and B lymphocytes, monocytes, and macrophages (41). However, HLA-E, transcribed in most tissues (24), can be mobilized to the cell surface by different peptides, such as stress protein peptides and peptides derived from different pathogens (16, 18). We thus performed assays in PBMC, as described in (37), as well as in primary epithelial cells.

Our data showed that incubation with an HLA-H signal peptide mobilized HLA-E at the cell surface of T-Lymphocytes, monocytes, B-Lymphocytes, and primary epithelial cells. The incubation time required for a significant effect was compatible with cell properties, as peak expression was reached after 4 h in antigen-presenting cells and after 16 h in primary epithelial cells.

We confirmed that, physiologically, PBMC from HLA-E*01:03 homozygous individuals had higher HLA-E cell surface expression than PBMC from HLA-E*01:01 homozygous individuals. Functional differences between the two isoforms, HLA-E^*^01:01 and HLA-E^*^01:03, which display similar frequencies (50%) in different populations (42), involve relative peptide affinity, cell surface expression, and potential lytic activity on NK cells (43).

Whether HLA-E isoforms associated with the HLA-H signal peptide display different tolerogenic activity remains to be explored. The higher affinity of the HLA-G-derived non-amer-HLA-E complex with a CD94/NKG2C receptor complex was reported and explained by the 10th amino acid (Phe) in the HLA-G signal peptide (44). In HLA-H, this amino acid was identical to HLA-A (Leu) and different from HLA-G.

The fact that *HLA-H* ^*^02:07 is so widespread and that mRNA expression is high in cells suggests that this HLA pseudogene deserves further investigation. Our results suggest that *HLA-H* may be functional. Many questions, however, need to be addressed: different expressions according to *HLA-H* alleles must be confirmed in primary tissues, particularly concerning the HLA-H*02:07 allele; *HLA-H* mRNA translation has to be explored, as our experiments were performed with a synthetic HLA-H signal peptide; and potential immune protection by HLA-E associated with an HLA-H signal peptide also has to be studied. Finally, supplementary studies will also be needed to investigate the existence and functional activity of a full-length HLA-H protein.

## Data Availability Statement

The datasets generated for this study are available on request to the corresponding author.

## Ethics Statement

The studies involving human participants were reviewed and approved by CERC-SFCTCV-2018-5-6-9-8-32-DjXa. The patients/participants provided their written informed consent to participate in this study.

## Author Contributions

FJ, DG, JP, and JD contributed to the conception and design of the study. JD organized the database. MD, DG, and JD performed the statistical analysis. JD wrote the first draft of the manuscript. All authors contributed to the acquisition, analysis, and interpretation of data for the work and contributed to manuscript revision, read and approved the submitted version.

### Conflict of Interest

JP was employed by company Xegen. The remaining authors declare that the research was conducted in the absence of any commercial or financial relationships that could be construed as a potential conflict of interest.

## Supplementary Material

Supplementary Figure 1 HLA-E, -G, and -H expression in HBEC (dCt: delta of cycle threshold, expression quantified by Q-PCR normalized by ACTB endogenous gene).

Supplementary Figure 2 HLA-H reads according to *HLA-H* deletion in RNA-sequencing data from the 1000 Genomes Project analyzed with the PolyPheMe software. Samples homozygous or heterozygous for HLA-H (all but *A***23/24*) are compared to samples hemizygous for *HLA-H* (*A***23/24*).

Supplementary Figure 3 HLA-H reads according to *HLA-H* deletion in HLA^*^01:01 samples in RNA-sequencing data from the 1000 Genomes Project.

Supplementary Figure 4 HLA-H reads according to *HLA-H* alleles in homozygous samples for HLA-H in RNA-sequencing data from the 1000 Genomes Project.

Supplementary Figure 5 HLA-H reads according to *HLA-H* alleles in samples bearing *H***02:01/H***02:01* vs. *H***02:01/H***02:07* in RNA-sequencing data from the 1000 Genomes Project.

Supplementary Figure 6 PBMC stained by HLA-E antibody compared with isotype control according to HLA-E genotype.

Supplementary Figure 7 HLA-E (3D12) expression in PBMC subtypes T-Lymphocytes (CD3), monocytes (CD14), and B-Lymphocytes (CD19) after 4 h of peptide incubation.

Supplementary Figure 8 HLA-E expression in HBEC after 16 h of peptide incubation.

Supplementary Table 1 Primer/probe designed for HLA-H Q-PCR assay.

Supplementary Table 2 Peptide sequences used in HLA-E expression assay.

## Abbreviations

HLA: Human Leukocyte Antigen
LTx: Lung Transplant patients
LD: Linkage Disequilibrium
CLAD: Chronic Lung Allograft Dysfunction
DSA: Donor Specific Antigen
NK: Natural Killer cells
PBMC: Peripheral Blood Mononuclear Cells
HBEC: Human Bronchial Epithelial Cell
EDTA: Ethylene Diamine Tetra Acetate-anticoagulated
ALI: Air-Liquid Interface
CMV: Cytomegalovirus; Mean Fluorescence Intensity
DMSO: Dimethylsulfoxyde.
