# Supplementary figures and images for: HLA-H: Transcriptional Activity and HLA-E Mobilization
Source: Front Immunol. 2020 Jan 17;10:2986. doi: 10.3389/fimmu.2019.02986 (PMC6978722; doi:10.3389/fimmu.2019.02986)

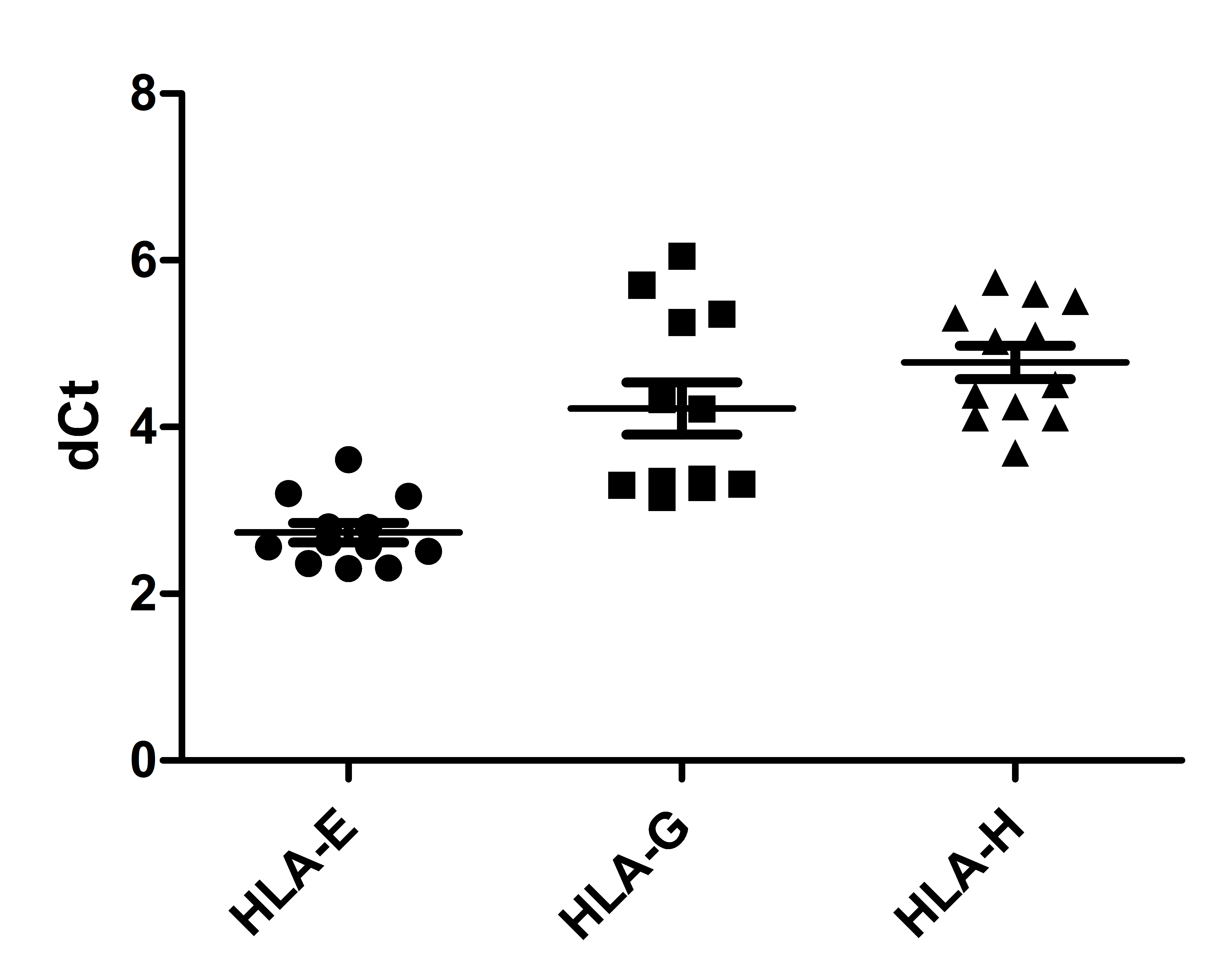

Supplement: Supplementary Figure 1 — HLA-E, -G, and -H expression in HBEC (dCt: delta of cycle threshold, expression quantified by Q-PCR normalized by ACTB endogenous gene). [file Image_1.jpg]

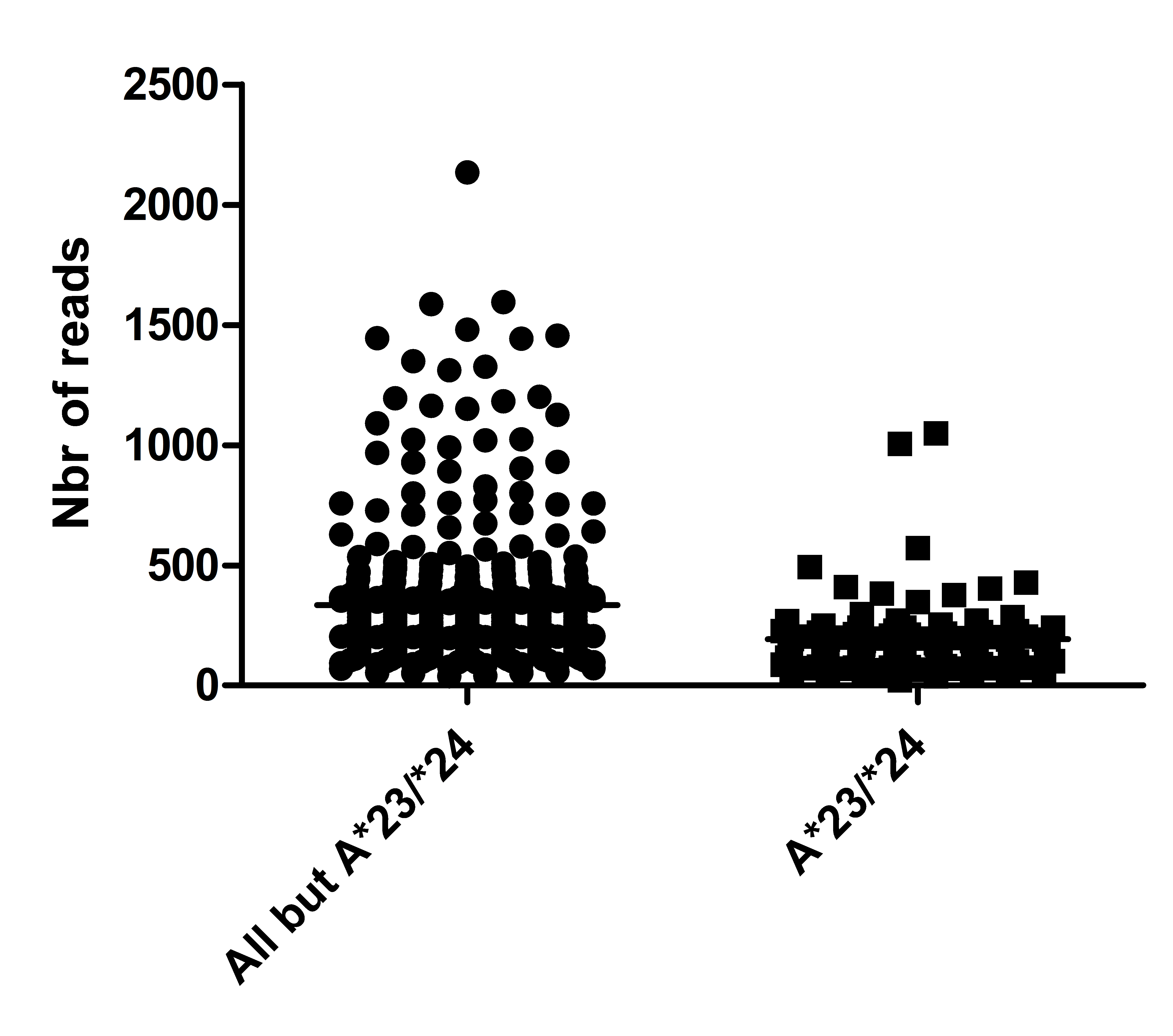

Supplement: Supplementary Figure 2 — HLA-H reads according to HLA-H deletion in RNA-sequencing data from the 1000 Genomes Project analyzed with the PolyPheMe software. Samples homozygous or heterozygous for HLA-H (all but A*23/24) are compared to samples hemizygous for HLA-H (A*23/24). [file Image_2.jpg]

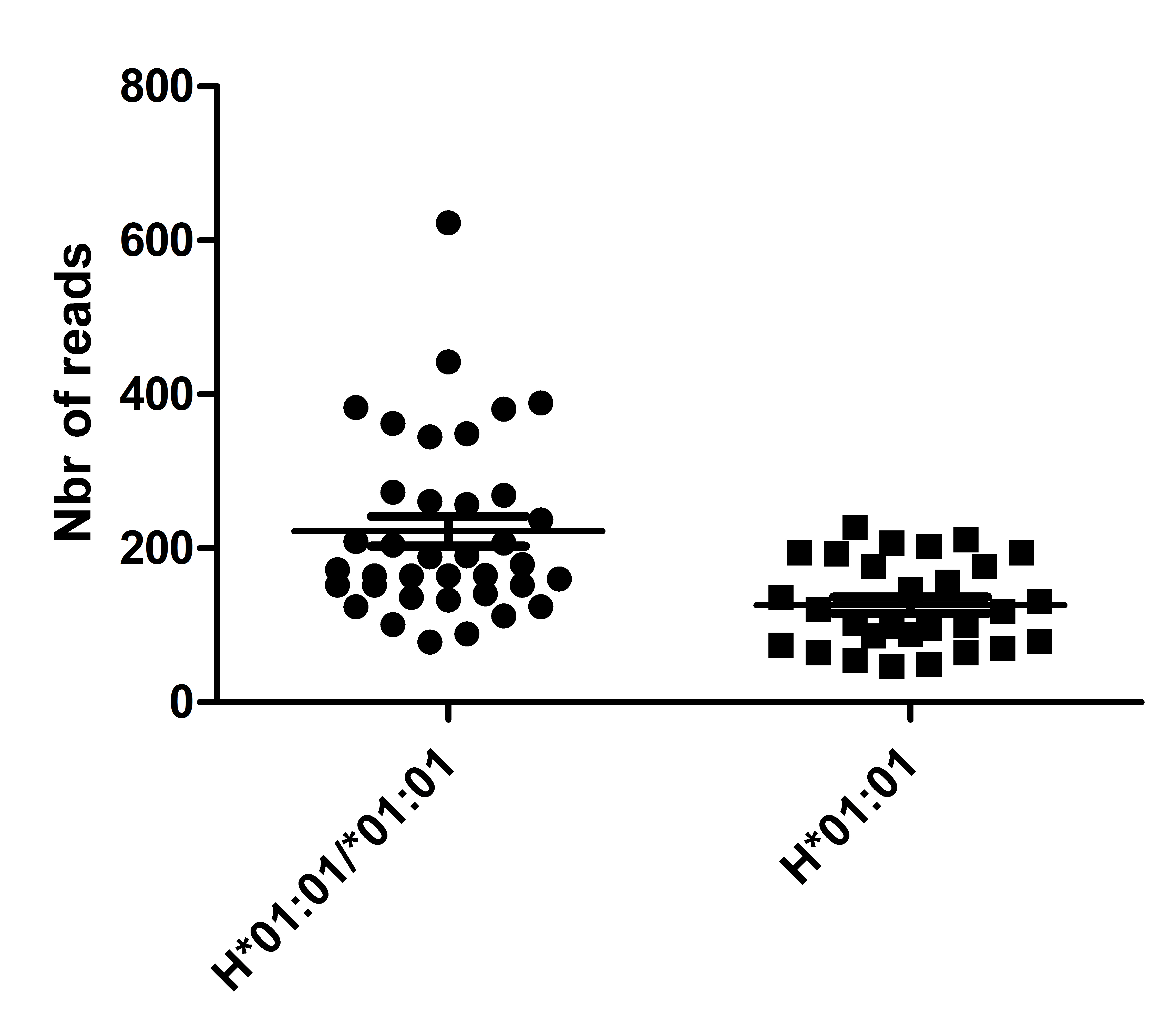

Supplement: Supplementary Figure 3 — HLA-H reads according to HLA-H deletion in HLA*01:01 samples in RNA-sequencing data from the 1000 Genomes Project. [file Image_3.jpg]

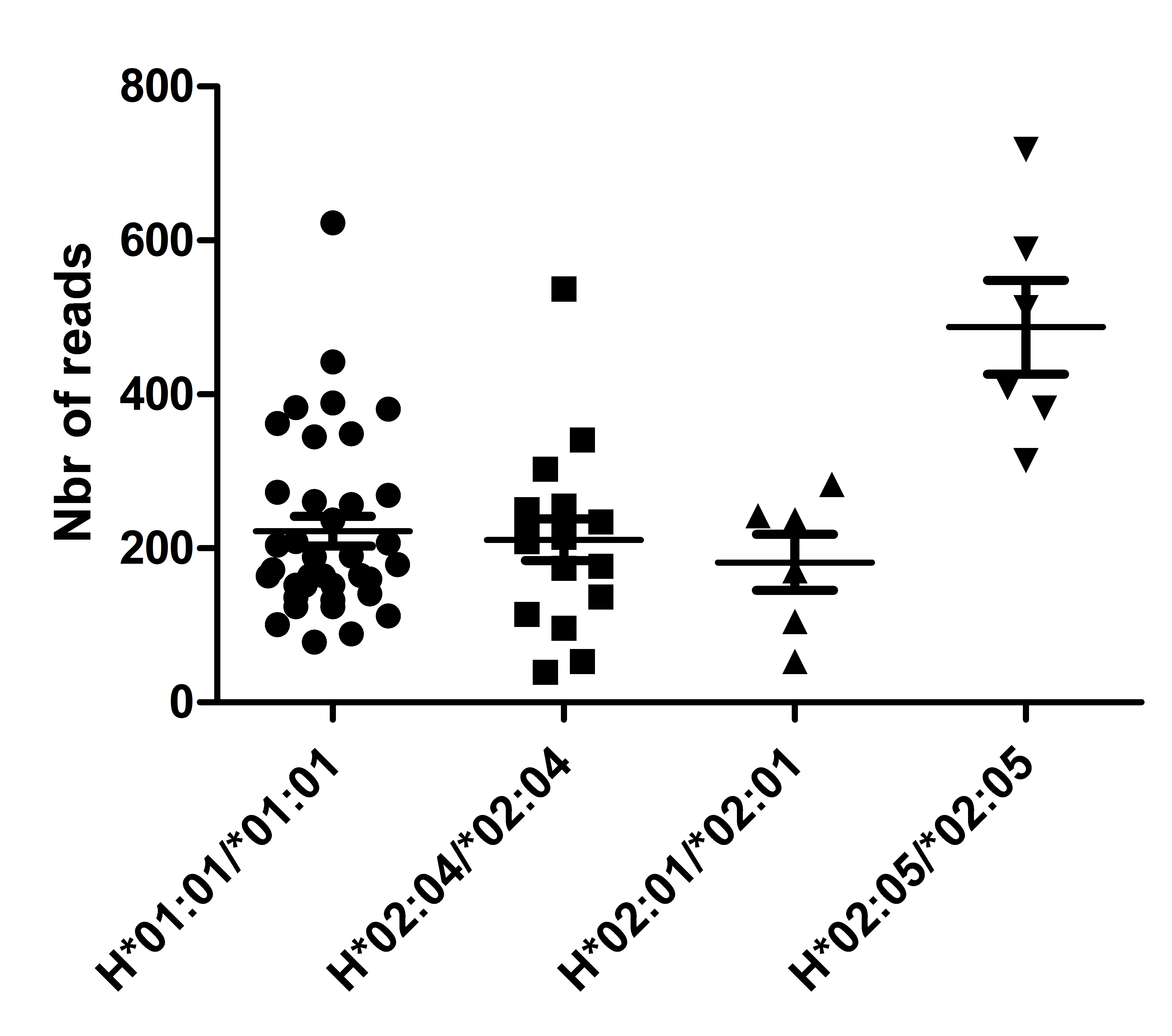

Supplement: Supplementary Figure 4 — HLA-H reads according to HLA-H alleles in homozygous samples for HLA-H in RNA-sequencing data from the 1000 Genomes Project. [file Image_4.jpg]

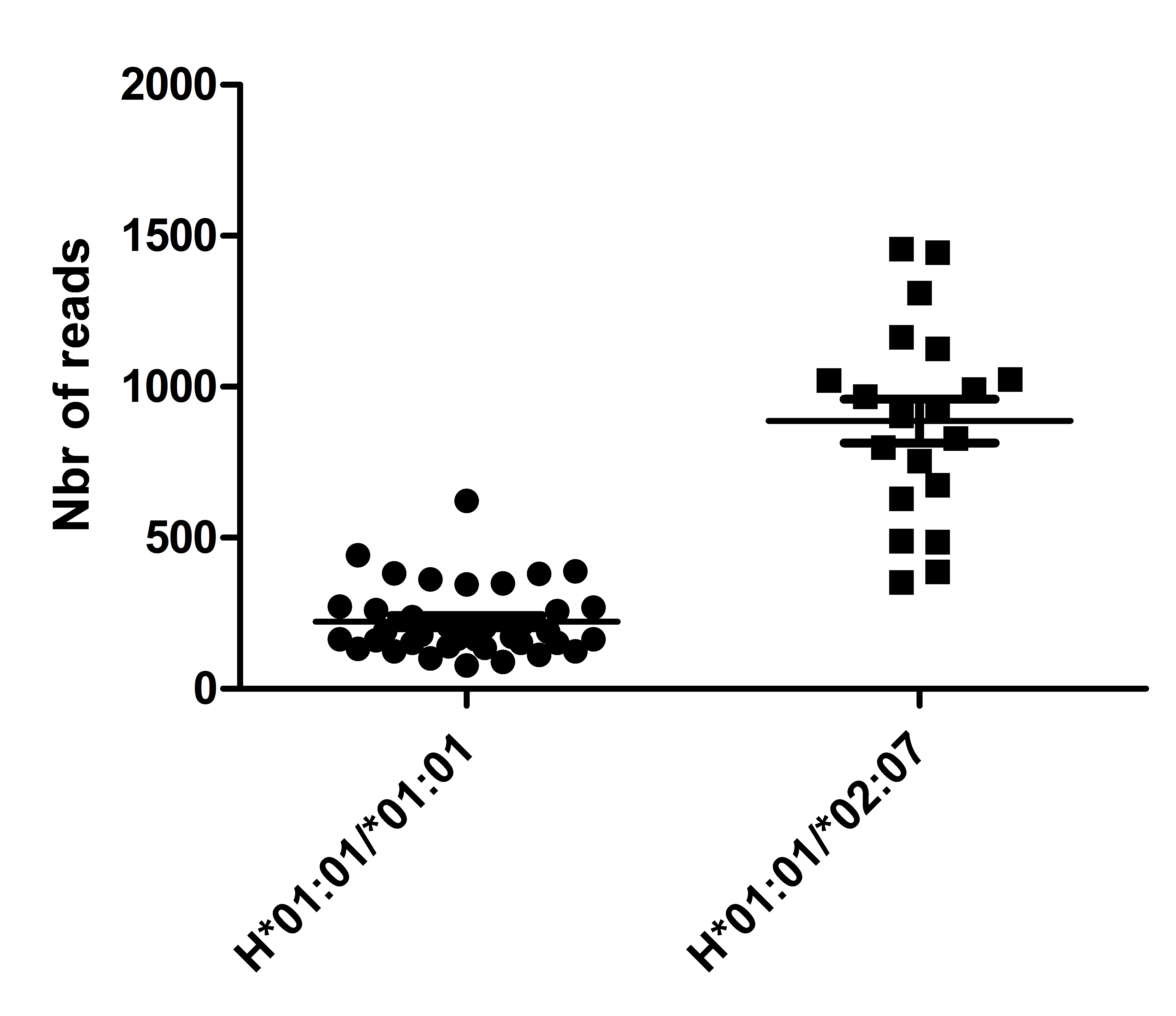

Supplement: Supplementary Figure 5 — HLA-H reads according to HLA-H alleles in samples bearing H*02:01/H*02:01 vs. H*02:01/H*02:07 in RNA-sequencing data from the 1000 Genomes Project. [file Image_5.jpg]

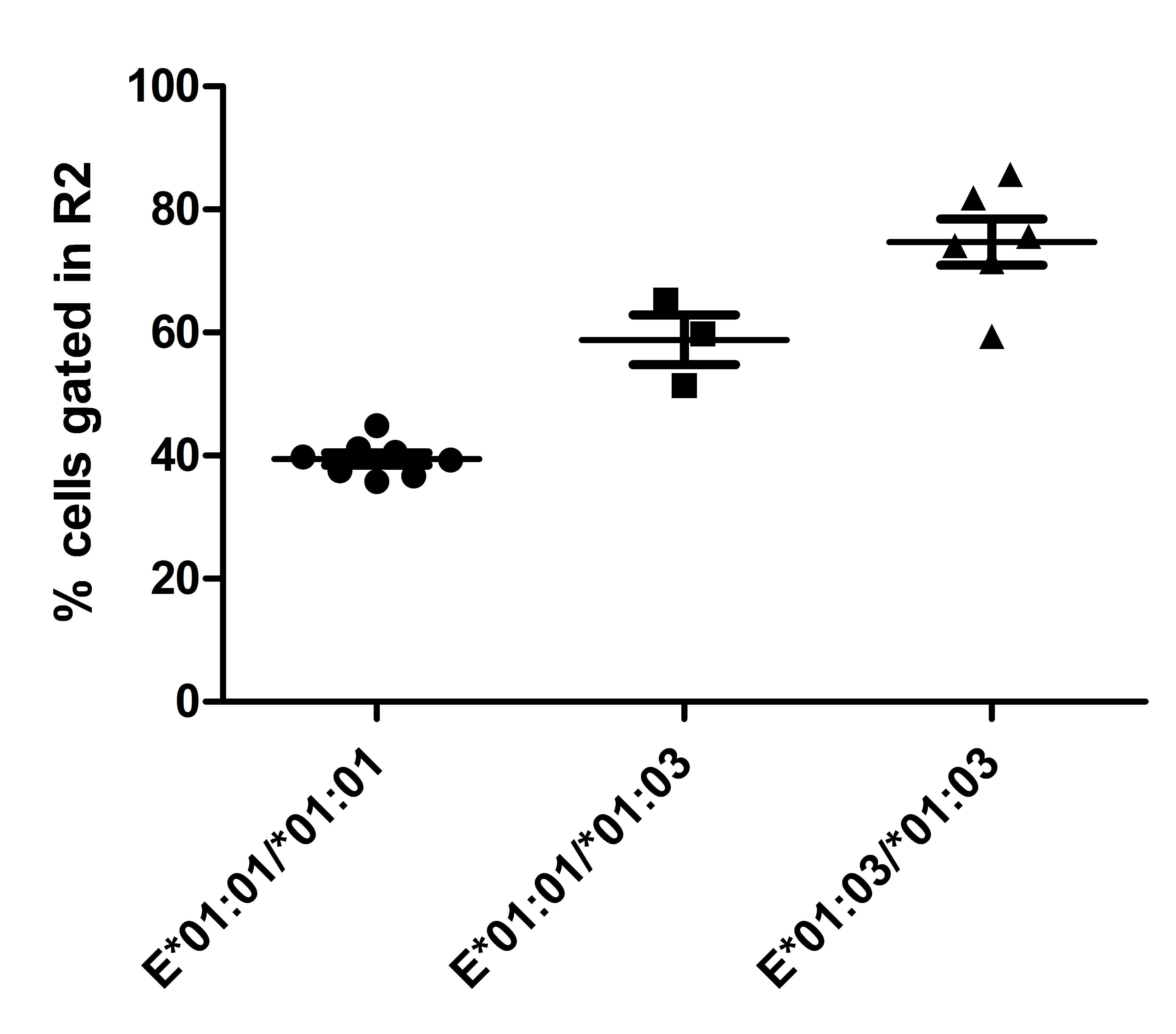

Supplement: Supplementary Figure 6 — PBMC stained by HLA-E antibody compared with isotype control according to HLA-E genotype. [file Image_6.jpg]

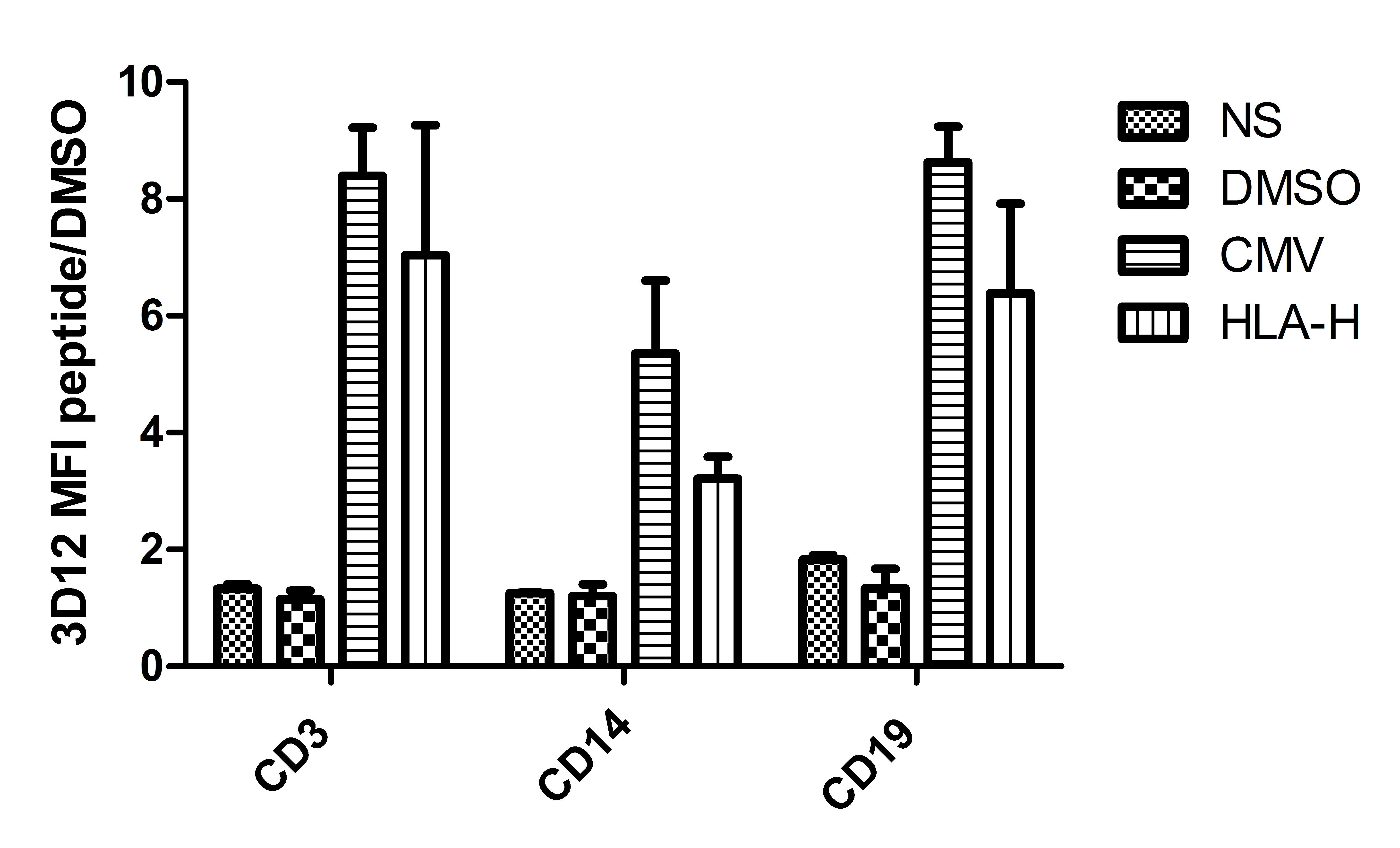

Supplement: Supplementary Figure 7 — HLA-E (3D12) expression in PBMC subtypes T-Lymphocytes (CD3), monocytes (CD14), and B-Lymphocytes (CD19) after 4 h of peptide incubation. [file Image_7.jpg]

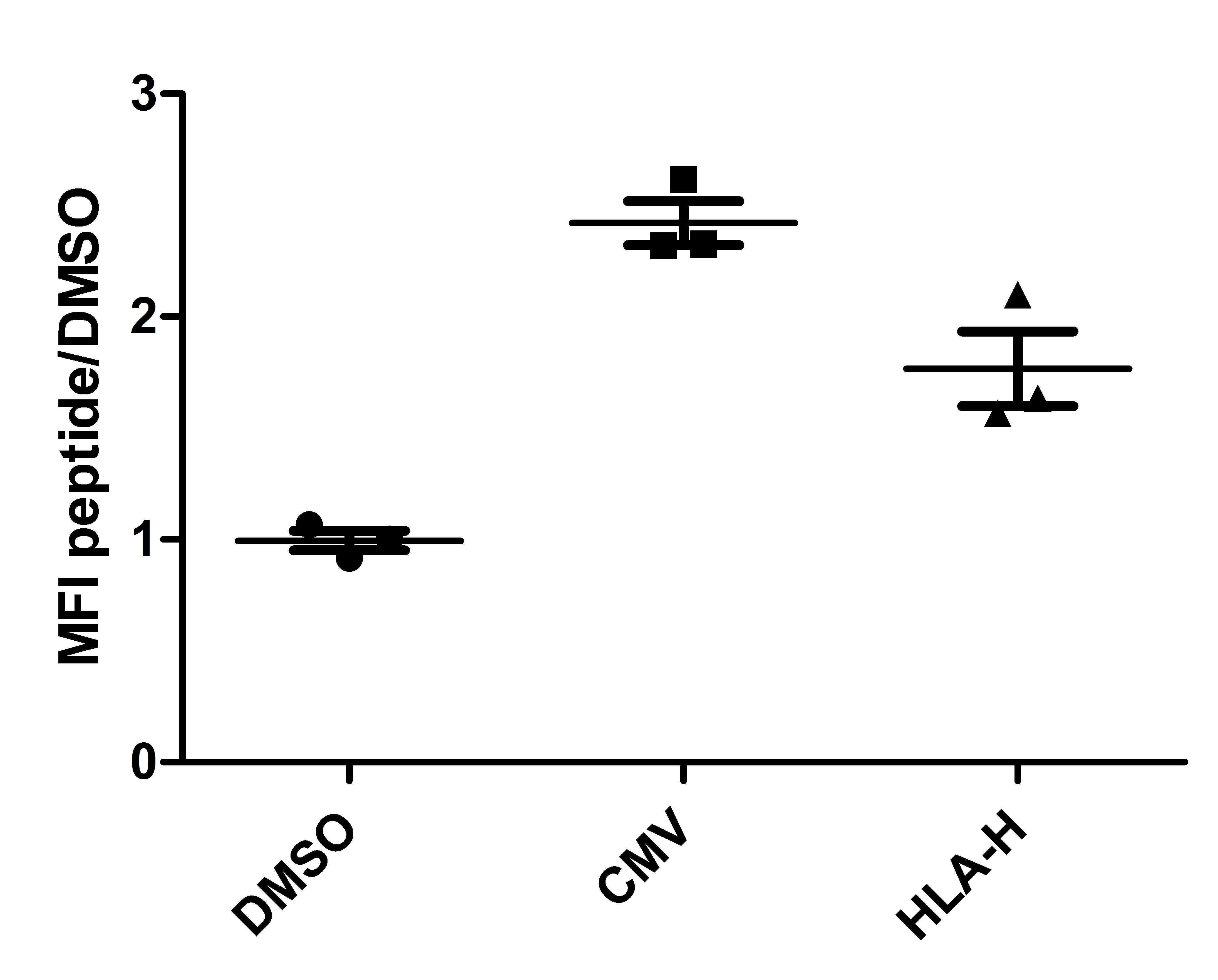

Supplement: Supplementary Figure 8 — HLA-E expression in HBEC after 16 h of peptide incubation. [file Image_8.jpg]
