# Supplementary material for: HLA-H: Transcriptional Activity and HLA-E Mobilization
Source: Front Immunol. 2020 Jan 17;10:2986. doi: 10.3389/fimmu.2019.02986 (PMC6978722; doi:10.3389/fimmu.2019.02986)
Supplement: Supplementary Table 1 — Primer/probe designed for HLA-H Q-PCR assay. [file Table_1.docx]

| **Primer/Probe** | **Sequence (5'>3')** | **HLA-H position**  **in CDS** |
| --- | --- | --- |
| Primer F (exon 1/2 junction) | TGGGCGC/GYTCCCACT | 73 |
| Primer R (exon 2) | GACGGAGATGAAGCGGGGCT | 147 |
| Probe (exon 2) | TATTTCTACACCACCATGTCCCGGC | 106 |

*Supplementary Table 1. Primer/probe designed for HLA-H Q-PCR assay*
