# Supplementary material for: HLA-H: Transcriptional Activity and HLA-E Mobilization
Source: Front Immunol. 2020 Jan 17;10:2986. doi: 10.3389/fimmu.2019.02986 (PMC6978722; doi:10.3389/fimmu.2019.02986)
Supplement: Supplementary Table 2 — Peptide sequences used in HLA-E expression assay. [file Table_2.docx]

| **Peptide** |  | **AA sequence** |
| --- | --- | --- |
| HLA-H |  | LMAPRTLLL |
| HLA-G |  | VMAPRTLFL |
| HLA-B15 |  | VTAPRTVLL |
| CMV |  | VMAPRTLVL |
| Neuromedin |  | GNLWATGHFM |

*Supplementary Table 2. Peptide sequences used in HLA-E expression assay.*
